# Recurrent genomic alterations in sequential progressive leukoplakia and oral cancer: drivers of oral tumorigenesis?

**DOI:** 10.1093/hmg/ddt657

**Published:** 2014-01-08

**Authors:** Nilva K. Cervigne, Jerry Machado, Rashmi S. Goswami, Bekim Sadikovic, Grace Bradley, Bayardo Perez-Ordonez, Natalie Naranjo Galloni, Ralph Gilbert, Patrick Gullane, Jonathan C. Irish, Igor Jurisica, Patricia P. Reis, Suzanne Kamel-Reid

**Affiliations:** 1 Division of Applied Molecular Oncology, Ontario Cancer Institute,; 2 Department of Pathology, Toronto General Hospital, Ontario Cancer Institute and; 3 Princess Margaret Cancer Centre and Techna Institute, University Health Network, Toronto, ON, Canada; 4 Department of Oral Diagnostics, Faculty of Dentistry (FOP), UNICAMP, Piracicaba, SP, Brazil; 5 Department of Laboratory Medicine and Pathobiology,; 6 Faculty of Dentistry and; 7 Departments of Medical Biophysics and Computer Science, University of Toronto, ON, Canada; 8 Prevention Genetics, Marshfield, WI, USA; 9 Department of Hematopathology, Vancouver General Hospital, Vancouver, BC, Canada; 10 Department of Pathology and Molecular Medicine, McMaster University, Hamilton, ON, Canada; 11 Department of Otolaryngology, Hospital Calderon Guardia, San Jose, Costa Rica; 12 Department of Otolaryngology/Surgical Oncology, Princess Margaret Hospital, University of Toronto and University Health Network, Toronto, ON, Canada; 13 Department of Surgery and Orthopedics, Faculty of Medicine, São Paulo State University (UNESP), Botucatu, SP, Brazil

## Abstract

A significant proportion (up to 62%) of oral squamous cell carcinomas (OSCCs) may arise from oral potential malignant lesions (OPMLs), such as leukoplakia. Patient outcomes may thus be improved through detection of lesions at a risk for malignant transformation, by identifying and categorizing genetic changes in sequential, progressive OPMLs. We conducted array comparative genomic hybridization analysis of 25 sequential, progressive OPMLs and same-site OSCCs from five patients. Recurrent DNA copy number gains were identified on 1p in 20/25 cases (80%) with minimal, high-level amplification regions on 1p35 and 1p36. Other regions of gains were frequently observed: 11q13.4 (68%), 9q34.13 (64%), 21q22.3 (60%), 6p21 and 6q25 (56%) and 10q24, 19q13.2, 22q12, 5q31.2, 7p13, 10q24 and 14q22 (48%). DNA losses were observed in >20% of samples and mainly detected on 5q31.2 (35%), 16p13.2 (30%), 9q33.1 and 9q33.29 (25%) and 17q11.2, 3p26.2, 18q21.1, 4q34.1 and 8p23.2 (20%). Such copy number alterations (CNAs) were mapped in all grades of dysplasia that progressed, and their corresponding OSCCs, in 70% of patients, indicating that these CNAs may be associated with disease progression. Amplified genes mapping within recurrent CNAs (*KHDRBS1*, *PARP1*, *RAB1A*, *HBEGF*, *PAIP2*, *BTBD7*) were selected for validation, by quantitative real-time PCR, in an independent set of 32 progressive leukoplakia, 32 OSSCs and 21 non-progressive leukoplakia samples. Amplification of *BTBD7*, *KHDRBS1*, *PARP1* and *RAB1A* was exclusively detected in progressive leukoplakia and corresponding OSCC. *BTBD7*, *KHDRBS1*, *PARP1* and *RAB1A* may be associated with OSCC progression. Protein–protein interaction networks were created to identify possible pathways associated with OSCC progression.

## INTRODUCTION

Head and neck squamous cell carcinomas (HNSCCs) are the sixth leading cause of cancer death worldwide (1,2). About one-fourth of all HNSCCs are oral squamous cell carcinomas (OSCCs), and are estimated >26 000 new cases and >5000 deaths in the USA every year (3) [Source: estimated new cases are based on 1995–2008 incidence rates from 47 states and the District of Columbia as reported by the North American Association of Central Cancer Registries (NAACCR), representing about 95% of the US population. Estimated deaths are based on US Mortality Data, 1994 to 2008, National Center for Health Statistics, Centers for Disease Control and Prevention.]. Patients with OSCC have benefited from the latest advances in surgical techniques, radiation therapy and chemotherapy, which help enhance quality of life and improve survival. Despite these advances, the 5-year survival rate of patients remains at ∼50% (4–6). Low survival rates are mainly due to the presence of late-stage disease at diagnosis and disease recurrence. In order to improve patient survival, more accurate methods of detection of lesions at a risk for malignant transformation and a better understanding of the genetic events associated with disease progression are needed. Since malignant transformation is due to genetic damage over time (7), the identification of genetic changes in sequential progressive lesions within the oral cavity is thus potentially useful for predicting lesions at a risk for malignant transformation.

It is known that a significant proportion (up to 62%) of OSCCs (8,9) arise from precursor oral potential malignant lesions (OPMLs), such as leukoplakia. Oral leukoplakia is a lesion that presents as a ‘white patch’ in the oral mucosa (9,10). Currently, these lesions are classified based on clinical and histopathological assessment. Clinically, leukoplakia lesions are homogeneous or non-homogeneous, the latter having a higher risk of transformation. Histologically, they are classified as non-dysplastic or dysplastic (10), and the presence of epithelial dysplasia is associated with an increased risk of malignant transformation of up to 31% (11). However, clinical and histological characteristics have limited prognostic value for predicting which leukoplakia will progress to malignancy.

Genetic biomarkers may be clinically useful to identify lesions at a risk for malignant transformation. Previous studies identified gains and losses of large chromosomal regions as well as loss of heterozygosity (LOH) associated with progression in dysplasias and OSCCs from different patients (12–14). As these studies identified fairly large genomic regions, important genes dominantly involved with OSCC progression remain unknown.

High-resolution global genomic profiling of sequential, progressive leukoplakia lesions and same-site invasive oral carcinoma from same patients enabled us to identify recurrent copy number gains and losses, and narrow down genes likely to be involved in progression of leukoplakia to invasive OSCC. Genes identified herein may represent prognostic markers, to identify leukoplakia lesions at a risk of progression. Assessment of such biomarkers could then be used to initiate early intervention, ultimately improving patient survival.

## RESULTS

We successfully applied a whole genomic amplification (WGA) protocol for amplification of low yield DNA from formalin fixed paraffin-embedded (FFPE) samples, to accurately assess DNA copy number gains and losses. Array comparative genomic hybridization (aCGH) using amplified DNA allowed the identification of global copy number gains and losses, with similar results when compared with DNA from unamplified FFPE samples; results showed a high correlation between copy number alterations (CNAs) identified in amplified versus unamplified DNA samples (*R*^2^ = 0.80–0.97).

**Figure 1.**
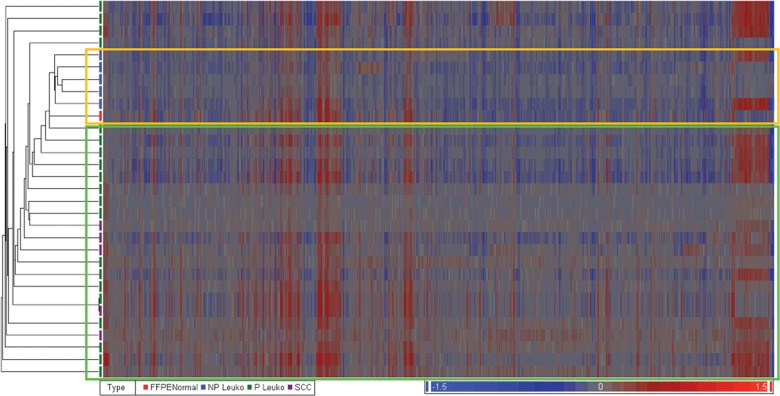
Unsupervised hierarchical clustering analysis of CNAs. Data were generated using Partek Genome Suite software. Two main distinct clusters were observed: normal/non-progressive leukoplakia (yellow box) and progressive leukoplakia/OSCCs (green box), demonstrating similarity between malignant and progressive lesions (purple and green, respectively), and showing how normal and non-progressive leukoplakia samples (red and blue, respectively) were clustered separately.

CNAs were analyzed blinded to sample histology. Unsupervised hierarchical clustering analysis showed that the majority of progressive leukoplakia lesions (16/20) and OSCCs clustered together, separately from normal and non-progressive leukoplakia samples (Fig. 1), indicating that progressive leukoplakia and OSCCs share common CNAs.

**Figure 2.**
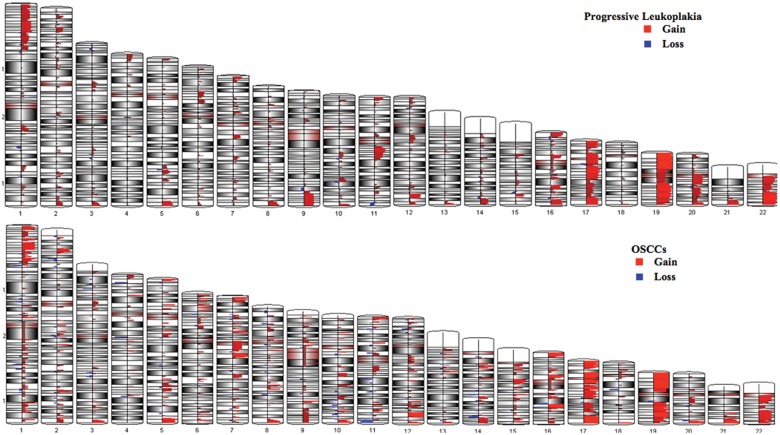
CNA profiles of progressive leukoplakia (upper panel) and OSCCs (bottom panel). CNA profiles were similar in progressive leukoplakia lesions and same-site OSCCs. Regions of gain (in red) are over-represented, compared with regions of loss (in blue).

The genomic segmentation algorithm used to detect amplifications and deletions showed a total of 8409 change calls in the group of progressive leukoplakia and OSCCs, and 2170 change calls in non-progressive samples. These results were then filtered for CNAs found in both groups that were very rare or absent among copy number variations (CNVs) found in the general population. This analysis showed that out of the 8409 change calls, 4081 (48.5%) were unique to progressive leukoplakia and same-site OSCCs; and that out of the 2170 change calls, 1146 were CNAs unique to non-progressive leukoplakia. These 1146 change calls present in non-progressive samples were then removed from the 4081 CNAs found within progressive leukoplakia and same-site OSCCs. Such an approach was used to accurately determine the genetic changes involved in oral cancer progression, since we selected CNAs specific to progressive leukoplakia and corresponding OSCCs and absent in non-progressive leukoplakia samples. This analysis showed a total of 2935 CNAs present in progressive leukoplakia and OSCCs, but not in non-progressive leukoplakia. A larger number of gains were common to progressive leukoplakia and OSCC (80%), in contrast to a small number of losses (20%) (Fig. 2). DNA losses were represented mainly on chromosomes 5q31.2 (35%), 16p13.2 (30%) and 9q33.1–9q33.2 (25%). The remaining DNA losses were found on chromosomes 17q11.2, 3p26.2, 18q21.1, 4q34.1 and 8p23.2 (20%) (Supplementary Material, Fig. S1A). DNA copy number gains were identified on chromosome 1p in 20/25 cases (80%) with high-level amplifications at 1p35 and 1p36. Amplifications were also found at 11q13.4 (68%), 9q34.13 (64%), 21q22.3 (60%) and 6p21 and 6q25 (56%). Other regions of chromosomal gain included 10q24, 19q13.2, 22q12, 5q31.2, 7p13 and 14q22 (48%) (Supplementary Material, Fig. S1B).

**Figure 3.**
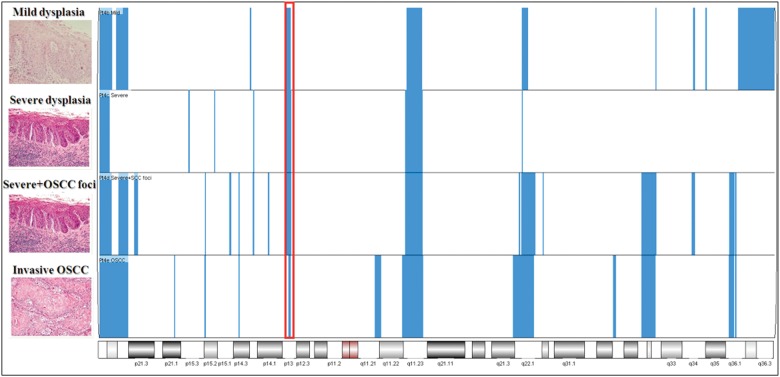
A representative example of CNA (7p13 gain) identified in sequential progressive samples of patient 4 (red box). 7p13 gains were detected in all progressive lesions from low to high grade sequential progressive leukoplakia and OSCCs, suggesting that 7p13 gains may be associated with disease progression. Other regions of 7p gains, detected in progressive samples from patient 4, were not highlighted as they were not present in all progressive leukoplakia from other patients.

We observed an average of 113, 61, 153 and 178 significant change calls (*P* < 0.001) in sequential progressive samples of mild (*n* = 4), moderate (*n* = 3), severe (*n* = 6) leukoplakia and OSCCs (*n* = 25), across all patients. Figure 3 shows a representative example of CNAs found in the sequential progressive samples of patient 4. Samples containing foci of cells with two different grades (e.g. samples 4d, 10d and 10e) were included in the group of higher grade of dysplasia. Also, as severe oral dysplasia and carcinoma *in situ* have very similar histology and do not differ biologically, they were considered a unique group in our analysis.

**Figure 4.**
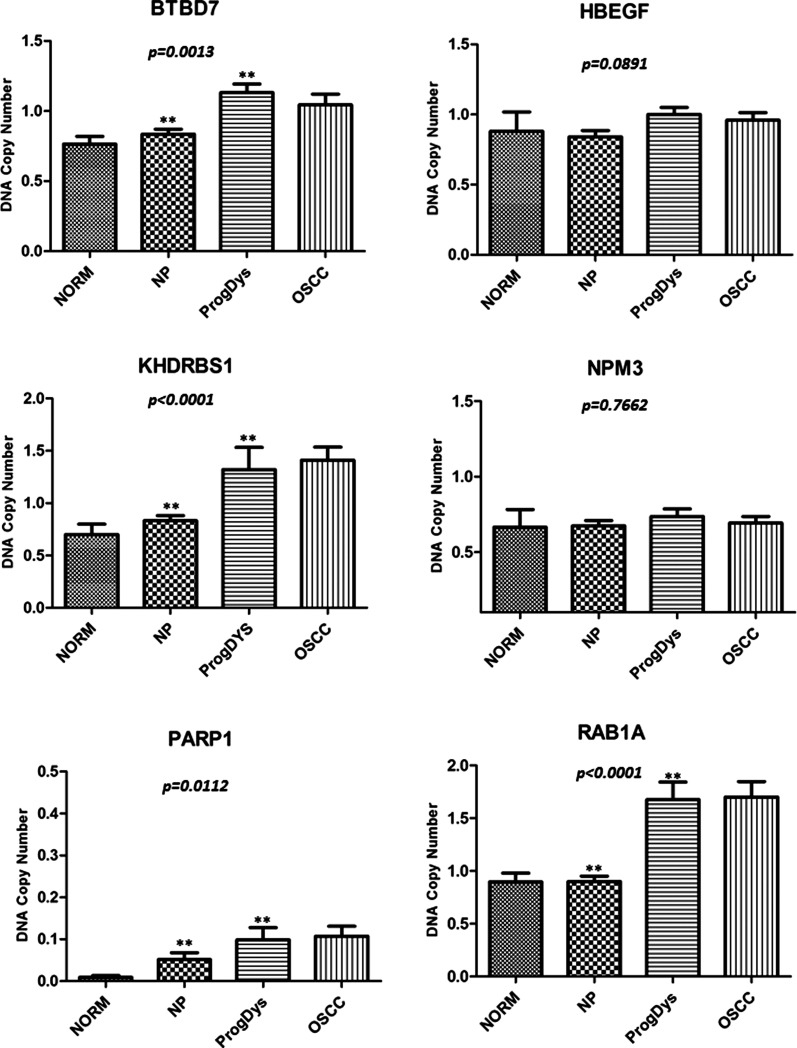
RQ-PCR validation of genes mapped within regions of gains, as identified by aCGH. DNA gains of *BTBD7*, *KHDRBS1*, *PARP1* and *RAB1A* were validated in an independent set of progressive leukoplakia and OSCCs compared with non-progressive leukoplakia samples. Asterisks indicate statistical significance for DNA copy number changes in progressive leukoplakia and OSCC compared with non-progressive leukoplakia and normal oral tissues (*P*-values are given by the Kruskal–Wallis test).

We detected a total of 696 different chromosomal regions, commonly altered in progressive leukoplakia and OSCCs; 552/696 were regions of gain and 144/696 were regions of loss. In order to determine the alterations involved in the progression of OPML to invasive carcinoma, we focused our analysis on CNAs from these 696 regions that were present in at least one OSCC and one preceding OPML from the same patient. This analysis revealed 193 regions of gains and 15 regions of loss. Notably, 38/193 gains and 5/15 losses were common to all sequential samples (OSCC and preceding leukoplakia lesions). This analysis confirmed recurrent losses mapped to 3p26.2, 8p23.2, 9q33.1–9q33.2, 17q11.2 and 18q21, and gains to 1q32, 1p35–36, 2p14, 5q31, 6p21, 6q25, 7p13, 10q24, 11q13.4, 12p13, 14q22, 19q13 and 22q12.3. These CNAs were detected in low to high grade dysplasias, and their corresponding OSCCs, for the majority (70%) of patients. Since these regions may contain genes that are relevant for the process of neoplastic transformation of leukoplakia to OSCC, we annotated 263 genes (255 amplified and 8 deleted), all mapped within the identified regions. We used the public databases UCSC Genome browser (http://genome.ucsc.edu/) and NCBI (http://www.ncbi.nlm.nih.gov/) as well as DAVID tool v6.7 (15,16) to search for and annotate the biological roles of these genes and their potential involvement in cancer biology. Based on these analyses, we selected 78 genes (Supplementary Material, Table S1), which were then subjected to further evaluation using ONCOMINE v.4 cancer profiling database (Research edition), a cancer microarray database and web-based data-mining platform aimed at facilitating discovery from genome-wide expression analyses (17). This analysis allowed us to verify whether deregulated mRNA expression of these 78 genes has been detected in independent samples from patients with head and neck cancer. Due to sample availability, given the rarity and small sample sizes of leukoplakia lesions, we were able to validate the top six genes from our analysis, which have also been described to play key roles in cancer-related pathways. Validation analysis was then performed in an independent cohort of progressive dysplasias as well as its corresponding OSCCs, and compared with a group of non-progressive leukoplakia lesions, using RQ-PCR (Fig. 4). *BTBD7, KHDRBS1*, *PARP1* and *RAB1A* were all found to be amplified in progressive leukoplakia lesions and OSSCs and not amplified in non-progressive leukoplakia. *NPM3* and *HBEGF* did not significantly differ between progressive and non-progressive leukoplakia.

A network-based analysis was used to map protein–protein interaction (PPI) networks of genes within CNAs commonly identified in progressive leukoplakia and OSCC. Such analysis may be useful to identify commonly disrupted genetic pathways in progressive leukoplakia and OSCC. PPI networks showed five proteins (HBEGF, KHDR1, PAIP2, PARP1 and RAB1A) interconnected through several common partner proteins; four of them shared the same partner protein (Supplementary Material, Fig. S2). Since we previously identified a 3-miRNA signature (hsa-miR-21, has-miR-345 and has-miR-281b) of oral leukoplakia progression using the same samples described herein for aCGH profiling (18), we sought to integrate PPI networks of genes and miRNAs. Using NAViGaTOR (19), we combined the network from Supplementary Material, Figure S2 with a network from proteins that correspond to targets of the 3-miRNA signature. This network-based integrative analysis identified regulatory networks that may be disrupted by CNAs during oral cancer progression. Our results showed that two miRNAs (hsa-miR-21 and hsa-miR-345) were highly interconnected and associated with three proteins (BTBD7, PARP1 and RAB1A) within the first network (Supplementary Material, Fig. S3). BTBD7 was linked to hsa-miR-345 and RAB1A was linked to hsa-miR-21, KHDR1 and PARP1, respectively. KHDR1, PARP1 and RAB1A were highly connected proteins within the PPI networks identified.

### Tobacco consumption data in patients with progressive versus non-progressive leukoplakia

We performed an additional analysis to assess whether history of tobacco consumption was associated with progressive leukoplakia. We found that tobacco smokers were the significant majority of patients with progressive leukoplakia and same-site OSCC (89.4%, 17/19 patients), compared with patients with non-progressive leukoplakia (*P* = 0.0051, 95%CI, Fisher's exact test). Most patients with non-progressive leukoplakia did not have a history of tobacco consumption (61, 5%, 8/13).

## DISCUSSION

aCGH remains a high-throughput molecular technique widely used to determine CNAs across the cancer genome. This technology has been applied to DNA extracted from archived FFPE clinical specimens in several tumor types to elucidate key genes involved in disease development and progression (20,21). In particular, genomic analysis of tumor DNA has identified alterations in sequence and copy number associated with diagnosis, prognosis and treatment response in several cancers (22–24). Amplification or deletion of distinct chromosomal regions can lead to deregulated gene expression, thus conferring a growth advantage to malignant cells (25). Amplified or deleted genes could, therefore, be important targets for therapeutic intervention, and identification of such CNAs may also help elucidate potential mechanisms of tumor development and progression.

Our study is the first to examine CNAs in sequential, progressive oral lesions from the same patients, with the aim of identifying CNAs associated with malignant transformation, as the tissue progresses from benign epithelium to carcinoma. Non-progressive samples were also examined to ensure that CNAs were exclusively detected in progressive and not in non-progressive leukoplakia, and would thus likely represent driver changes associated with malignant transformation*.* Such changes provide targets for functional validation of molecular mechanisms of disease progression.

When we compared CNA profiles of histologically different tissues using unsupervised hierarchical clustering analysis, we were able to show that non-progressive leukoplakia segregated from progressive leukoplakia and invasive OSCCs. CNAs identified mainly increased from the lowest to the highest grade of dysplasia, culminating with invasive OSCCs, which contained the largest number of changes. Our data demonstrate that progressive leukoplakia lesions already possess many of the genetic alterations present in invasive cancers; this is consistent with the hypothesis that the majority of genetic alterations occur at an early phase during head and neck cancer progression (26). Leukoplakia lesions may thus have genetic instability, as it has been shown that chromosome instability is detected in the majority (9/10) of aneuploid OSCCs that arose from aneuploidy leukoplakia (27).

Tobacco smoking, an important risk factor associated with OSCC development, can lead to genomic instability and may help promote transformation of pre-malignant lesions in the oral cavity (28–30). Indeed, we showed that tobacco as well as alcohol consumption was associated with progressive lesions. Screening of patients with OPML who have history of tobacco and alcohol consumption may thus be important for early detection of oral cancer, ultimately improving patient survival (30).

Herein, we detected genomic instability with deletions on 3p, 9q and 18q in at least 20% of progressive leukoplakia and same-site OSCC. Allelic losses of 3p and 9p have been reported in HNSCC from different anatomic sites (31,32); 18q loss has been associated with poor prognosis and metastasis in patients with HNSCC (33,34). LOH on 9p, combined with *TP53* mutations, have been recently associated with malignant transformation of oral leukoplakia, and suggested as biomarkers of progression (35).

Over-representation of CNAs identified at lp35–36, 11q13, 19q and 22q12, in low-to-high-grade sequential progressive leukoplakia and OSCC, agrees with a previous study showing that these regions were correlated with HNSCC progression (31). In particular, 11q13 gains/amplification have been associated with poor prognosis of patients with HNSCC (36). Gains at 11q have been associated with a higher risk of esophageal squamous cell carcinoma development; notably, 11q13.1–13.4 is one of the most gene-rich regions on 11q, showing DNA gains correlated with increased RNA expression in >80% of the genes mapped on this minimal region (37). Additionally, 11q13 gains were correlated with poor prognosis of patients with other tumors, such as prostate (38) and thyroid (39), larger tumor size in hepatocellular carcinoma (40), and were suggested as predictive markers of distant recurrence in patients with breast cancer (41).

Our aCGH data analyses narrowed down 16 altered genes within regions containing CNAs associated with progressive leukoplakia and same-site invasive OSCC. Significantly amplified genes (*BTBD7*, *CAMSAP1L1*, *CHRDL2*, *FBXO7*, *GMPK2*, *HBEGF*, *IRF9*, *KHDRBS1*, *NPM3, PAIP2*, *PARP1*, *RAB1A*, *REC8* and *TBRG4*) and significantly deleted genes (*CSMD1* and *MYO5B*) were detected in progressive leukoplakia and OSCCs, and not in non-progressive lesions. Gene ontology (GO) categories for those genes include functions associated with protein binding, cell cycle, cell differentiation, cell proliferation, transcription factors and cell surface receptors linked to signal transduction (42) (Supplementary Material, Table S2). The most representative GO category comprised protein binding genes, which included *BTBD7*, *FBXO7*, *IRF9*, *PAIP2*, *PARP1*, *REC8* and *MYO5B*. The deleted gene, *CSMD1*, encodes a membrane–membrane interaction protein. *RAB1A* is involved in protein transportation and transduction signaling, and *KHDRS1, PARP1* and *IRF9* are transcription factor regulators. Additionally, *TBRG4* is involved in apoptosis; *CHRDL2* is a key player in cell differentiation, and *HBEGF* is part of the epidermal growth factor receptor/transduction signaling gene category.

Genetic alterations in sequential OPMLs and same-site OSCCs may represent key driver changes in disease development. In particular, DNA amplification can occur at an earlier stage of cancer development, in which oncogenic pathways may be critically disrupted (43). One of the most frequently (80%) amplified gene in our data set was *KHDRBS1* (also known as *p62*), a cell proliferation and cell surface receptor of signal transduction located at 1p35.1. The signaling adaptor p62 is induced by RAS, with p62 levels increased in human tumors, and required for RAS-induced survival and cellular transformation (44). Considering that the balance between cell death and survival is important in oncogenic transformation processes, assessment of *KHDRBS1-RAS* in oral cancer progression may be relevant to further elucidate the molecular mechanisms of oral tumorigenesis. Interestingly, *RAB1A*, a member of the RAS oncogene family, which maps to 2p14, was significantly amplified in progressive leukoplakia and OSCC from our data set. *RAB1A* overexpression has been previously identified in human tongue squamous cell cancer and suggested as a biomarker of tongue carcinogenesis (45).

Another amplified gene was *HBEGF*, mapped at 5q31.3. This gene encodes a protein that is an EGFR ligand up-regulated in diverse pathological conditions, including cancer (46,47). Interestingly, *HBEGF*, together with two other genes, *COX2* and *ST6GALNAC5*, mediate breast cancer cell passage through the blood-brain barrier (46). In addition, *COX-2* overexpression has been shown in oral cancer and high-risk oral lesions (48). Recently, a significant increase in COX-2 protein expression has been detected in moderate dysplastic oral leukoplakia when compared with inflammatory fibrous hyperplasia lesions and suggested to be associated with early stages of oral tumorigenesis (49).

*EGFR* overexpression is associated with poor prognosis of patients with HNSCC, and its inhibition improves patient survival (50). Increased EGFR-DNA copy number has been associated with OSCC development in patients having a precursor, same-site leukoplakia, which also over-expressed EGFR. EGFR inhibitors may thus be potentially useful in preventing malignant transformation of such leukoplakia lesions (51).

Additionally, *EGFR* is one of the most frequently amplified and highly expressed gene in both human and mouse oral tumors (*P* < 0.01) (52). Of note, *RAB1A* is a downstream effector of *EGFR*. Since amplification of *EGFR* and other oncogenes may coexist in single cells of oral dysplasia (43), cells may thus be dependent on multiple oncogenes for OPML progression.

The roles of the *BTBD7, NPM3* and *PARP1* genes in tumorigenesis have also been described (53–55), although to our knowledge, *BTBD7* and *NPM3* have not been previously examined in oral tumorigenesis. *BTBD7* has been suggested to play a role in epithelial cell dynamics and branching morphogenesis, by inducing *SNAIL2* and suppressing E-cadherin expression; such events alter cell morphology and reduce cell-to-cell adhesion (56). Interestingly, podoplanin, a small mucin-type transmembrane protein that modulates molecular pathways of cell migration and invasion (57), is over-expressed in dysplastic oral leukoplakia and associated with grade of dysplasia and risk of progression to cancer (58).

Other cancer-associated genes identified in our aCGH experiments include *CHRDL2*, mapped on 11q13.4. *CHRDL2* encodes a putative extracellular matrix protein and was shown to be over-expressed in breast, lung and colon tumors, compared with corresponding normal tissues (59). *REC8*, located at 14q12, is part of the cohesin gene family. REC8 protein participates in the segregation of homologs at the first meiotic division (60), and *REC8* up-regulation determines the extent of arrested mitoses and polyploidy in lymphoma cell lines (61). *REC8*, together with *PAIP2*, are differentially expressed in OSCCs (62) and involved in cellular mitosis and translation-related activities. Translation initiation is regulated in response to mitogenic stimulation, and thus associated with cell cycle progression and cell growth. Combined over-expression of the cell-cycle related proteins TP53/p16(INK4a) as well as the proliferation marker Ki-67 was suggested as a marker of malignant transformation and able to classify high-risk leukoplakia (63). Expression changes in the components of the translational machinery can lead to global changes, such as an increase in protein expression and translational activation of mRNA and miRNA molecules that control cell growth and proliferation. Although translational control alterations occur in cancer, further investigation is required to determine their role in tumor development and progression (64).

Other studies have identified large chromosomal regions and LOH events in dysplasias and OSCCs, but did not characterize such changes in sequential, progressive samples from same patients (12–13,43,65). Additionally, CNA-associated genes have not been previously characterized into PPI networks and pathways. Herein, PPI network analysis showed that proteins encoded by *HBEGF*, *KHDRBS1*, *PAIP2*, *PARP1* and *RAB1A* are interconnected through several common partner proteins within a common network; most genes within this network have functions associated with genome maintenance, cellular fate and organization and transcriptional control.

In the previous study (18), we identified a 3-miRNA signature associated with the progression of oral leukoplakia to same-site OSCC. We showed that increased expression levels of hsa-miR-21, hsa-miR-181b and hsa-miR-345 were significantly associated with increased lesion severity during progression of leukoplakia to OSCC. Since changes in miRNA expression may occur through several mechanisms, such as transcriptional or post-transcriptional regulation, and changes in the expression of miRNA biogenesis enzymes; such mechanisms may be partially attributed to genomic gains or losses. Herein, aCGH global genomic approach allowed us to integrate CNAs associated with sequential progressive lesions from different patients, with the previously identified 3-microRNA signature (miR-21, miR-181b and miR-345) associated with leukoplakia progression, in the same samples. Interestingly, both miR-345 (14q32.2) and miR-181b (1q32.1) map to regions of DNA gain identified by aCGH in progressive leukoplakia and OSCC, suggesting that over-expression of these two miRNAs may be due to gains at these genomic loci. CNAs were absent in the chromosomal region where miR-21 maps, suggesting that in progressive leukoplakia and OSCC, miR-21 could be regulated by other transcriptional and/or post-transcriptional changes. A network-based integrative analysis can be used to identify pathways that may be disrupted by CNAs, including miRNA-mRNA targets, during disease progression. Using PPIs, our study is highly suggestive of a connection between specific CNAs, miRNAs and gene expression, thus shedding light on our understanding of leukoplakia to OSCC progression. Recently, higher expression of miR-21, miR-181b or miR-345 in leukoplakia has been associated with cytological features used to grade leukoplakia; e.g. increased number of mitotic figures, increased nuclear to cytoplasmic ratio or hyperchromasia, nucleoli number and size (66). Interestingly, miR-181 over-expression has been reported to enhance lymph-node metastasis in OSCC through cell migration (67).

A consistent pattern of changes on selected chromosome arms enabled us to identify specific genes involved in the OSCC progression. Potential genomic markers of interest were identified on chromosomes 1p, 2p, 5q, 8p, 11q, 14q, 18q and 22q and may represent drivers involved in oral cancer progression. By integrating data on CNAs and the previously identified 3-miRNA expression signature, we were able to show a possible interaction between these changes; these may represent alterations to genomic and post-transcriptional control mechanisms of specific genes and pathways important for disease progression. Functional studies directly targeting these genes/pathways will help clarify their role in the progression of leukoplakia to OSCC.

## MATERIALS AND METHODS

### Patient samples

#### Training sample set

All patient samples were FFPE tissues. We collected 30 samples from 10 patients. Of these, 25 were sequential samples from 5 patients (20 progressive leukoplakia and 5 same-site carcinomas); therefore, all carcinomas had at least one corresponding premalignant oral leukoplakia. Of the 20 leukoplakia lesions, 4 were non-dysplastic and 16 were dysplastic (mild, moderate or severe). The remaining five samples were non-progressive leukoplakia lesions, from five patients. Training sample set characteristics are detailed in Table 1. A commercial normal genomic DNA (Promega, Madison, WI, USA) was used as control in the aCGH experiments.

**Table 1.** Training sample set characteristics: sequential oral leukoplakia and same-site OSCCs

Patient	Sample ID	Site	Histopathological diagnosis	Group	Date of biopsy (month/year)	Age	Gender	Tobacco
1	1a	Tonsil	Focal keratosis^^a^^	PL	10.2001	83	F	Yes
	1b	Alveolus + FOM	Mild squamous hyperplasia^^a^^	PL	07.2003			
	1c	Anterior FOM	Carcinoma *in situ*	PL	05.2004			
	1d	Anterior FOM	Invasive moderately differentiated OSCC	OSCC	05.2004			
4	4a	Tongue	Severe squamous dysplasia	PL	11.1997	40	M	No
	4b	Tongue	Mild squamous dysplasia	PL	10.1998			
	4c	Tongue	Severe squamous dysplasia	PL	11.1998			
	4d	Tongue	Severe dysplasia + SCC micro foci	PL	11.1998			
	4e	Tongue	Invasive moderately differentiated OSCC	OSCC	02.2000			
5	5a	Right lateral tongue	Keratosis^^a^^	PL	03.1993	65	F	Yes
	5b	Right lateral tongue	Moderate dysplasia	PL	03.1993			
	5c	FOM	Severe dysplasia	PL	10.1994			
	5d	Buccal mucosa	Mild dysplasia	PL	02.1997			
	5e	Left buccal mucosa	Invasive OSCC	OSCC	10.1997			
10	10a	Left buccal mucosa	Keratosis^^a^^	PL	03.1991	60	M	Yes
	10b	Left buccal mucosa	Moderate dysplasia	PL	09.1995			
	10c	Left buccal mucosa	Mild dysplasia	PL	09.1996			
	10d	Left buccal mucosa	Moderate + Severe dysplasia	PL	10.2003			
	10e	Left buccal mucosa	Severe dysplasia + carcinoma *in situ*	PL	11.2003			
	10f	Left buccal mucosa	Invasive moderately differentiated OSCC	OSCC	06.2004			
15	15a	Right tongue	Mild dysplasia	PL	04.1994	82	F	Yes
	15b	Right tongue	Moderate dysplasia	PL	04.1994			
	15c	Right tongue	Keratosis mild atypia	PL	02.2001			
	15d	Right tongue	Moderately differentiated OSCC	PL	10.2003			
	15e	Right tongue	Mild squamous hyperplasia^^a^^	OSCC	03.2004			
1NP		Mandible gingiva	Mild dysplasia	NPL	1997	32	M	No
2NP		Mandible lingual mucosa	Mild dysplasia	NPL	2000	58	M	No
3NP		Buccal mucosa	Moderate dysplasia	NPL	2001	49	M	No
4NP		FOM	Moderate dysplasia	NPL	2001	60	M	No
5NP		FOM	Severe dysplasia	NPL	2001	63	F	No

PL, progressive leukoplakia; NPL, non-progressive leukoplakia; OSCC, oral squamous cell carcinoma; FOM, floor of mouth; WD, well differentiated; MD, moderately differentiated; PD, poorly differentiated; F, female; M, male.

^a^Non-dysplastic lesions.

### DNA isolation from FFPE samples

All samples underwent histopathological analysis by an experienced oral pathologist (G.B.) to ensure the presence of dysplasia or carcinoma in at least 80% of each tissue section. Samples were needle macro-dissected to select the target cell population for DNA extraction and genomic analysis. In short, genomic DNA was isolated from 5 to 10 (10 μm thick) FFPE tissue sections. After xylene deparaffinization, tissues were incubated in Cell Lysis Solution buffer (5 PRIME, Gaithersburg, MD, USA) and Proteinase K solution (20 mg/ml) for 2 days at 56°C (fresh aliquots of proteinase K were added at 17 and 24 h). Genomic DNA was isolated and purified using the ArchivePure DNA Cell/Tissue Kit-4g (5 PRIME), with final elution into sterile ddH_2_O. DNA samples were quantified using a NanoDrop Spectrophotometer, and checked by agarose gel electrophoresis for quality. All samples yielded DNA of sufficient quantity and quality for analysis. Samples within the training set were subjected to WGA using a Sequenase-based approach (modified from the Affymetrix Chromatin Immunoprecipitation Assay, as per Sadikovic *et al*. (68), in order to yield enough DNA quantity for aCGH analysis (∼2 μg). High-quality normal male genomic DNA (Promega) was used as the reference sample, as described in other aCGH studies (20,69,70). Male genomic DNA (Promega) was heat fragmented for 10 min at 95°C, subsequently subjected to WGA and hybridized against each test sample.

### Sequenase-based WGA

Two rounds of WGA were used to randomly amplify the 30 FFPE DNA samples from the training set (minimum of 10 ng and maximum of 200 ng of DNA). This amplification protocol has been successfully used by others to amplify <10 ng of DNA and was utilized in the comparison of relative enrichment between two samples (71). The protocol comprises of two sets of enzymatic reactions (Supplementary Material, Table S3); in Round I, the sequenase enzyme is used to extend randomly annealed primers (Primer A) and to generate templates for subsequent PCR. During Round II, the specific primer B (the sequence of which is partially identical to Primer A) was used to amplify the templates previously generated by dNTP (10 mm) incorporation. Following each amplification round, DNA was purified using the QIAquick® PCR Purification Kit (Qiagen, Valencia, CA, USA), according to the manufacturer's protocol. The final purified PCR product was eluted into 50 µl of Sigma water, and 5 μl of product was used to run a 1% agarose gel, to verify the presence of a 200 bp–1 kb DNA ‘smear’ for successfully amplified samples.

To verify the fidelity of the WGA, we first sought to determine the correlation of data resulting from amplified and unamplified template DNA, by analyzing matched fresh-frozen and FFPE tumor samples from a same patient. In all experiments, WGA protocol was used for both test (tumor) and reference (Promega DNA) samples. Correlation data are given in Supplementary Material, Table S4. All WGA samples displayed adequate signal-to-background ratios. Array CGH profiles of paired samples did not display any chromosomal gains or losses due to WGA. Overall, we obtained consistent data when comparing amplified versus unamplified FFPE samples, resulting in high Pearson's correlation coefficient ranging between *R*^2^ = 0.80–0.97 for both samples (29T and 201T). These values were reflected in the mean absolute deviations of the log2 ratios, calculated for all probes across the genome on the array.

### Whole genome tiling array CGH

We used the NimbleGen 385K whole genome tiling v2.0 array, which contains over 385 000 oligonucleotides probes (60-mer with a median probe spacing of ∼7 kb) providing genome wide coverage. Array CGH experiments including quality control, DNA labeling, hybridization, scanning and data extraction were performed by NimbleGen Systems core facility (Reykjavik, Iceland). The complete experimental protocol is provided in the NimbleGen Arrays User's guide (https://projects.cgb.indiana.edu/download/attachments/5363/NimbleGen_CGH_Users_Guide_v3p1.pdf?version=2). Briefly, 1 µg of genomic DNA was used for dual color labeling (inverse Cy3/Cy5). All 30 samples were successfully labeled, meeting quality control criteria. Following hybridization, washing and scanning were performed according to the manufacturer's protocol (NimbleGen-Roche). Array CGH data generation was performed using commercially available software (SignalMap version 1.8, Nimblegen).

### aCGH copy number data analysis

Partek Genomics Suite (PGS) software was used to identify CNAs. First, the .*pair* data files were loaded into the PGS software, which automatically loaded log2 ratio intensities for all probes across the tiling array. We performed unsupervised hierarchical clustering analysis using Euclidean distance, average linkage, agglomerative method (PGS), blinded to sample identity. We first sought to identify CNAs associated with oral cancer progression. For this, we performed copy number analysis across all samples: progressive leukoplakia lesions with corresponding OSCCs (*n* = 25), and non-progressive leukoplakia samples (*n* = 5). We examined CNAs present in progressive leukoplakia and corresponding OSCCs, and absent in non-progressive leukoplakia. We then compared these data against CNV frequency data available from the general control population [2115 predominantly European background individuals; half from Ontario (19), and half from Germany (20)]. This analysis filtered out any CNVs present in the general population, which are not relevant to disease biology/tumorigenesis. Additionally, to map the genetic alterations occurring during progression, we assessed CNAs within the progressive samples from each patient.

CNAs were identified using the genomic segmentation algorithm in PGS. Genomic aberrations were assessed with a segmentation stringency of 10 consecutive genomic markers utilizing *P* < 0.001 as cut off, and a signal-to-noise ratio cut-off of 0.3 for amplifications and deletions. We used a copy number cut-off of two copies to identify gains and losses; ratios < 0.85 are considered regions of loss, whereas ratios >1.15 represent regions of gain. This analysis excluded genes mapped on sex chromosomes, and regions with no known genes.

### Validation of genes by real-time quantitative PCR (RQ-PCR)

An independent cohort of 49 patient samples (21 non-progressive leukoplakia, 28 paired progressive leukoplakia lesions and OSCCs) was used for validation of CNAs identified by aCGH. Patient samples characteristics are described in Supplementary Material, Table S5. DNA was isolated from FFPE samples as discussed previously. We annotated and identified 16 altered genes, which were represented by several probes (at least 10) within the regions of recurrent gains and losses. Of these, 14 genes, including *BTBD7*, *CAMSAP1L1*, *CHRDL2*, *GMPK2*, *FBXO7*, *HBEGF*, *IRF9*, *KHDRBS1*, *NPM3, PAIP2*, *PARP1*, *RAB1A*, *REC8* and *TBRG4* were amplified, and 2 genes *CSMD1* and *MYO5B* were deleted in progressive leukoplakia lesions and paired OSCCs, compared with Promega normal gDNA, but not in non-progressive samples (Supplementary Material, Table S6). We further selected 6 of these 14 genes (*BTBD7*, *HBEGF*, *KHDRBS1*, *PAIP2*, *PARP1* and *RAB1A*), which were mapped within CNA regions exclusively amplified in all sequential progressive leukoplakia lesions and OSCCs and not amplified in non-progressive leukoplakia samples, for validation by RQ-PCR. RQ-PCR was performed using TaqMan assays (Life Technologies, Foster City, CA, USA); the detailed protocol is shown in Supplementary Material, Table S7. Primer/probe sequences are available upon request. Amplification conditions were: 50°C for 2 min.; 95°C for 10 min.; 35 cycles at 95°C for 15 s followed by 60°C for 1 min. RQ-PCR was performed using the Applied Biosystems Gene Amp PCR System 9700 thermocycler. Analysis was performed using the Delta-Delta Ct method (72). Statistical analysis was performed in GraphPad Prism v5.01 using the non-parametric Kruskal–Wallis test and Fisher's exact test. The significance level was *P* < 0.05. DNA copy number was normalized to Promega DNA control.

### Protein–protein interaction network and pathways analyses

Genes selected for validation (*BTBD7*, *HBEGF*, *KHDRBS1*, *PAIP2*, *PARP1* and *RAB1A*) were mapped into corresponding proteins to generate PPI networks using I^2^D ver. 2.0 (http://ophid.utoronto.ca/i2d) (73,74) and visualized using NAViGaTOR ver. 2.14 (http://ophid.utoronto.ca/navigator) (19). We further integrated three previously identified miRNAs (hsa-miR-21, hsa-miR-181, hsa-345) (18) into PPI networks, in order to identify whether genes and miRNAs shared common regulatory networks. This was achieved by first mapping miRNAs to target genes using microRNA Data Integration Portal ver.1 (http://ophid.utoronto.ca/mirDIP) (75), and then mapping resulting genes to proteins and interaction networks in I2D. We integrated predicted mRNA targets using mirDIP with the aim of identifying other potential partner proteins into regulatory networks, which may contribute to leukoplakia progression. In addition, The Database of Annotation, Visualization and Integrated Discovery (DAVID) v6.7 was used for functional annotation of genes (15,16).

## SUPPLEMENTARY MATERIAL

Supplementary Material is available at *HMG* online.

*Conflict of Interest statement*. None declared.

## FUNDING

This work was supported by the Galloway Fund administered through the University Health Network (R.G. and S.K.R.), by the Cancer Research Society (Canada) (S.K.R.), and by Ontario Research Fund (GL2-01-030) (I.J.). Computational resources were supported in part by grants from the Canada Foundation for Innovation (#12301, #203373, #29272, #225404) and IBM (I.J.). Funding to pay the Open Access publication charges for this article was provided by OICR (Ontraio institute for cancer research), The Galloway Research Fund PMH Foundation, The Cancer Research Society and The Ontario MOHLTC.

## Supplementary Material

Supplementary Data
