# Supplementary material for: Recurrent genomic alterations in sequential progressive leukoplakia and oral cancer: drivers of oral tumorigenesis?
Source: Hum Mol Genet. 2014 Jan 8;23(10):2618–28. doi: 10.1093/hmg/ddt657 (PMC3990162; doi:10.1093/hmg/ddt657)
Supplement: Supplementary Data [file supp_23_10_2618__index.html]

Recurrent genomic alterations in sequential progressive leukoplakia and oral cancer: drivers of oral tumorigenesis? — Recurrent genomic alterations in sequential progressive leukoplakia and oral cancer: drivers of oral tumorigenesis? — Supplementary Data 

# Recurrent genomic alterations in sequential progressive leukoplakia and oral cancer: drivers of oral tumorigenesis?

## Supplementary Data

Supplementary Data

**Files in this Data Supplement:**

- Supplementary Data - Doc file
- Supplementary Table 1 - xls file
- Supplementary Table 2 - xls file
- Supplementary Table 3 - doc file
- Supplementary Table 4 - doc file
- Supplementary Table 5 - doc file
- Supplementary Table 6 - doc file
- Supplementary Table 7 - doc file
