# Supplementary material for: Recurrent genomic alterations in sequential progressive leukoplakia and oral cancer: drivers of oral tumorigenesis?
Source: Hum Mol Genet. 2014 Jan 8;23(10):2618–28. doi: 10.1093/hmg/ddt657 (PMC3990162; doi:10.1093/hmg/ddt657)

**Supplemental Figure 1.** (**A)** Chromosomal view segmentation for chromosomes 3, 5, 8, 9, 17, and 18 with significant regions of loss (blue circles) common between progressive leukoplakia and OSCCs. (**B)** Panel showing chromosomal view segmentation for chromosomes 1, 5, 6, 7, 10, 11, and 14 with significant regions of gains (red circles) across all samples. Heat Map indicates CNA < 2 copies in blue for loss, and > 2 copies in red for gains.


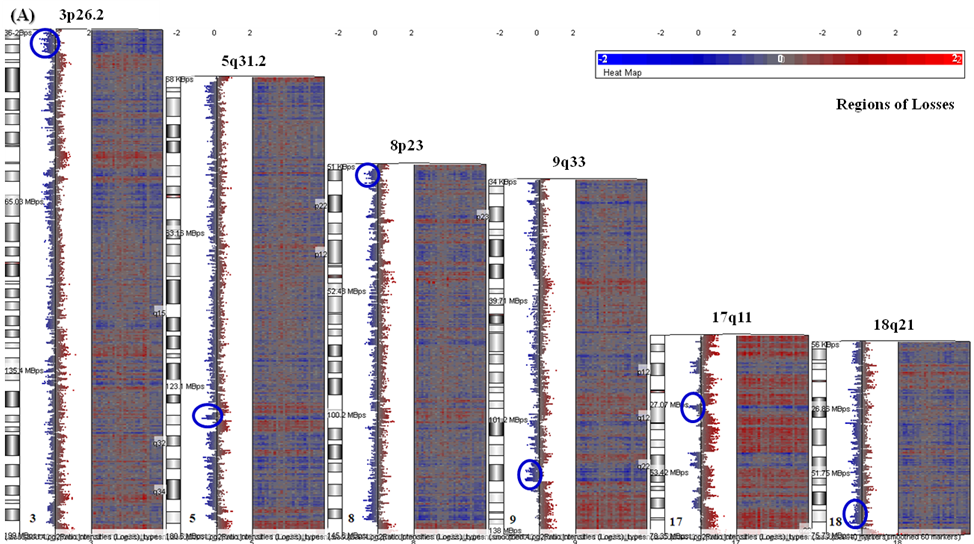


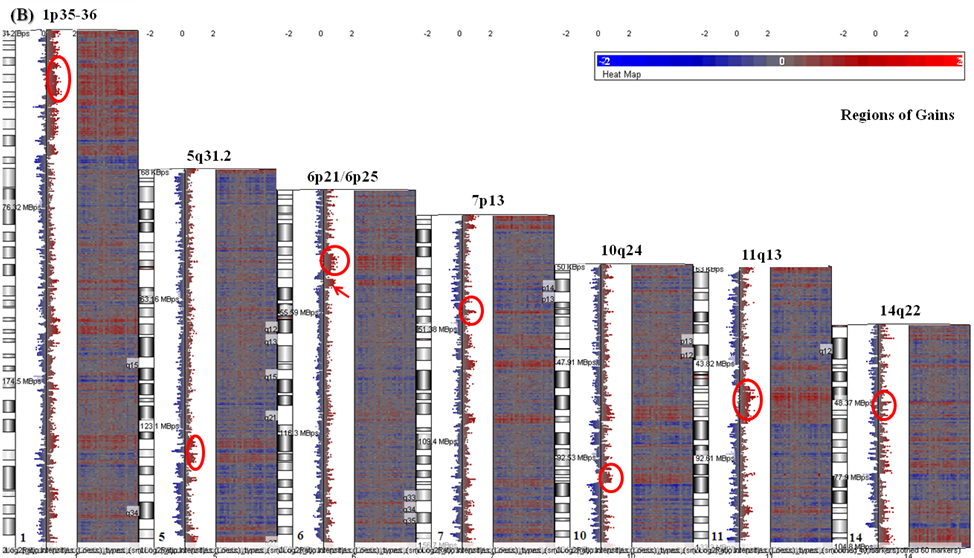


**Supplemental Figure 2.** Representative protein-protein interaction networks showing HBEGF, KHDR1, PAIP2, PARP1, and RAB1A interconnected through several common partner proteins.

**Supplemental Figure 3.** Integration of PPI networks of genes mapped within regions of CNAs and a previously identified 3-miRNA signature (hsa-miR-21, has-miR-345 and has-miR-281b) in the same samples profiled by aCGH. The panel shows that hsa-miR-21 and hsa-miR-345 are highly interconnected and associated with proteins encoded by *BTBD7*, *RAB1A* and *PARP1*. BTBD7 is linked to hsa-miR-345 and RAB1A is linked to hsa-miR-21, PARP1 and KHDRBS1, respectively. KHDRBS1, PARP1 and RAB1A are highly interconnected proteins.


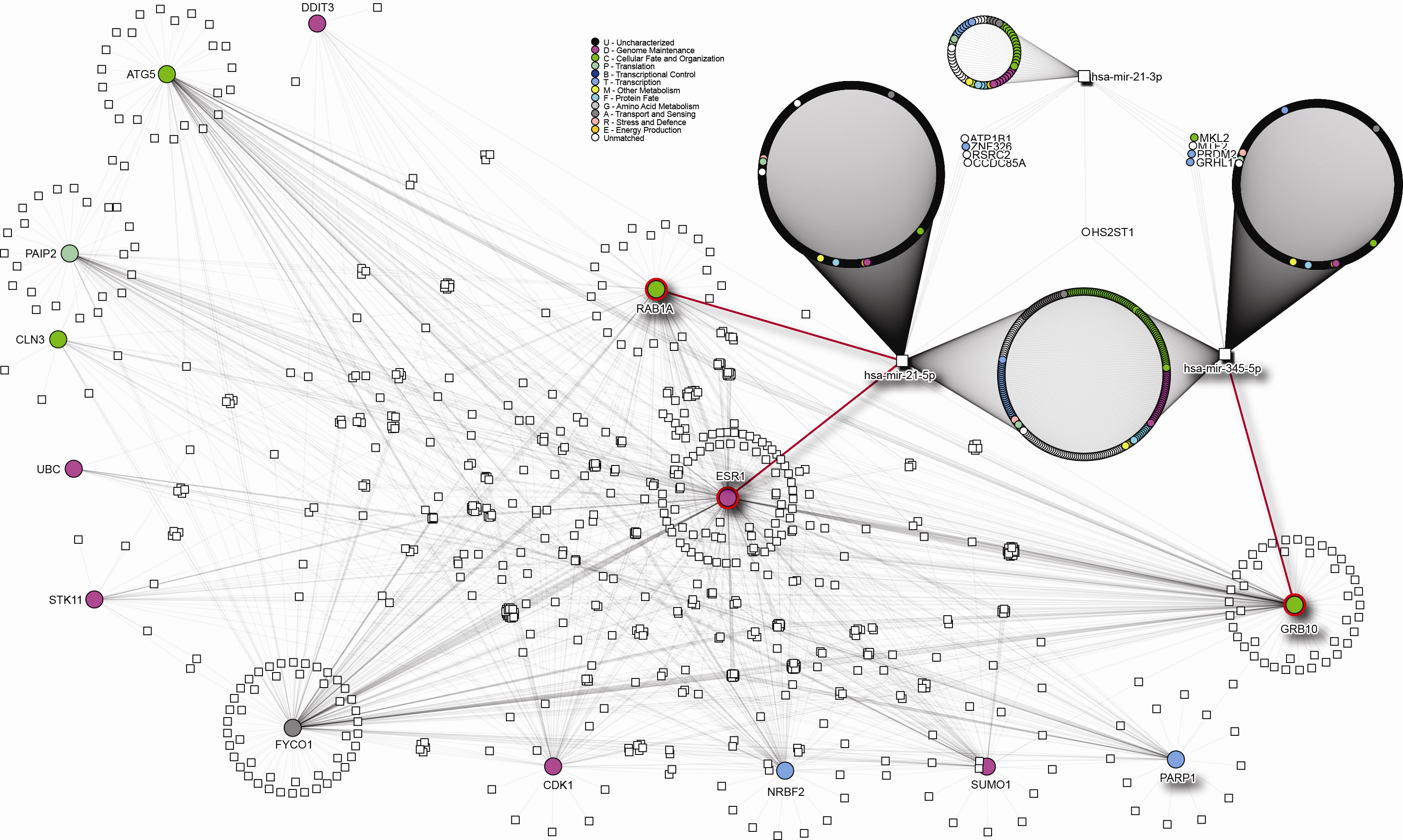


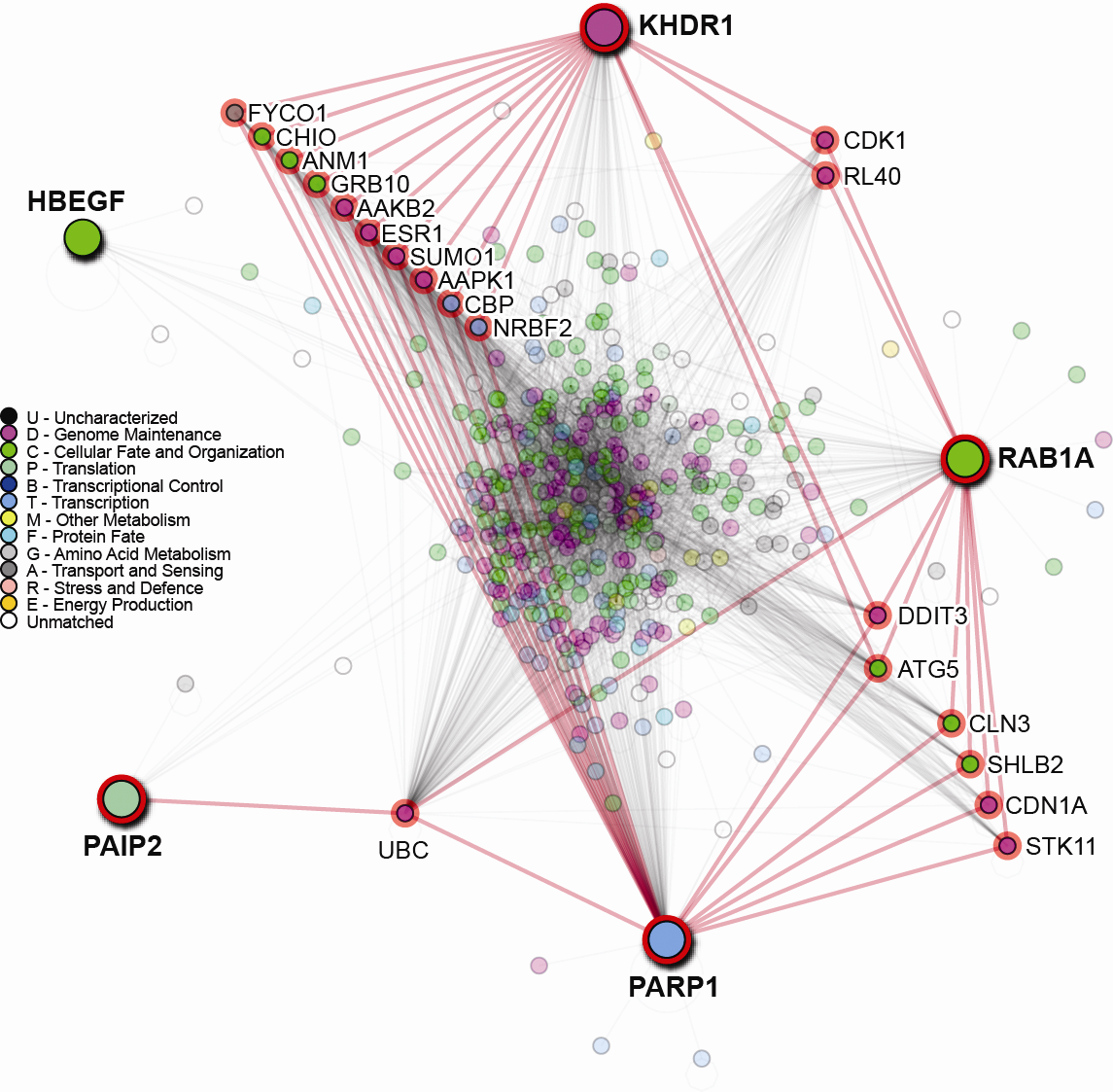

Supplement: Supplementary Data [file supp_ddt657_ddt657supp.doc]
