# Supplementary material for: Recurrent genomic alterations in sequential progressive leukoplakia and oral cancer: drivers of oral tumorigenesis?
Source: Hum Mol Genet. 2014 Jan 8;23(10):2618–28. doi: 10.1093/hmg/ddt657 (PMC3990162; doi:10.1093/hmg/ddt657)
Supplement: Supplementary Data [file supp_ddt657_ddt657supp_table3.doc]

**Supplemental Table 3.** PCR conditions for the two round whole genomic DNA amplification (modified from Affymetrix Chromatin Immunoprecipitation Assay, as per Sadikovic *et al.* JBC 2008).

| **Round I** | | |
| --- | --- | --- |
| **Components** | **Volume for 1Rxn** | **Cycling conditions** |
| DNA (10-200 ng) | 10 µL | 1. Heat 2 min at 94^o^C |
| 5x Sequenase Buffer | 4 µL | 2. Rapid cool to 10^o^C and hold 5 min at 10^o^C |
| Primer A (200 µM) | 4 µL | 3. Add reaction mixture* to sample |
|  |  | 4. Ramp from 10^o^C to 37^o^C over 8 min |
|  |  | 5. Hold at 37^o^C for 8 min; rapid ramp to 94 ^o^C and hold for 2 min. |
|  |  | 6. Rapid ramp to 10 ^o^C and hold for 5 min at 10 ^o^C while adding 1.2 μL of diluted Sequenase (1:10) |
|  |  | 7. Go back to step 4 (3 times) |
| **Round II** | | |
| Purified Round I template | 8 µL | 1. 2 min at 94 ^o^C |
| MgCl_2_ (50 mM) | 2.5 µL | 2. 1 min at 94 ^o^C |
| 10x PCR Buffer | 5 µL | 3. 2 min at 52 ^o^C |
| dNTP (10 mM) | 2 µL | 4. 3 min 72 ^o^C |
| Primer B (100 µM) | 2 µL | 5. Go to 2, 4 times |
| Taq Pol (5 U/µL) | 1 µL | 6. 30 sec 94 ^o^C 30 cycles |
| ddH_2_O | 29.5 µL | 7. 2 min at 52 ^o^C |
|  |  | 8. 1.5min at 72 ^o^C |
|  |  | 9. Go to 6, 24 times |
|  |  | 10. 5 min at 72 ^o^C |
|  |  | 11. ∞ at 4 ^o^C |

*Reaction Mixture: diluted sequenase (1/10 from 13 U/µl stock), 0.5 µL 25 mM dNTPs, 1 µL 0.1 M DTT and

0.1 µL 20 mg/ml BSA (total volume = 2.6 µL).
