# Supplementary material for: Recurrent genomic alterations in sequential progressive leukoplakia and oral cancer: drivers of oral tumorigenesis?
Source: Hum Mol Genet. 2014 Jan 8;23(10):2618–28. doi: 10.1093/hmg/ddt657 (PMC3990162; doi:10.1093/hmg/ddt657)
Supplement: Supplementary Data [file supp_ddt657_ddt657supp_table4.doc]

**Supplemental Table 4**. Correlation coefficients of aCGH profiling between unamplified *vs.* amplified DNA from FFPE tumor (T) samples from two patients.

| Chromosome | HN00 29T(unamplified) | HN02 201T(unamplified) |
| --- | --- | --- |
|  | HN00 29T (amplified) | HN02 201T (amplified) |
| 1 | 0.8812 | 0.7163 |
| 2 | 0.8734 | 0.7831 |
| 3 | 0.9125 | 0.6154 |
| 4 | 0.8452 | 0.7546 |
| 5 | 0.8321 | 0.7287 |
| 6 | 0.7542 | 0.5835 |
| 7 | 0.7743 | 0.6036 |
| 8 | 0.8438 | 0.7845 |
| 9 | 0.8125 | 0.7524 |
| 10 | 0.7904 | 0.6785 |
| 11 | 0.8562 | 0.7247 |
| 12 | 0.8693 | 0.8015 |
| 13 | 0.7742 | 0.7248 |
| 14 | 0.6623 | 0.5863 |
| 15 | 0.6321 | 0.5740 |
| 16 | 0.7597 | 0.6378 |
| 17 | 0.8452 | 0.7356 |
| 18 | 0.8631 | 0.7025 |
| 19 | 0.9523 | 0.8672 |
| 20 | 0.9729 | 0.8965 |
| 21 | 0.8735 | 0.8472 |
| 22 | 0.7582 | 0.7242 |
| X | 0.6834 | 0.6736 |
| Y | 0.7129 | 0.6895 |

Pearson’s correlation coefficients are given as a mean for all probes on the array, subdivided by chromosome.
