# Supplementary material for: Recurrent genomic alterations in sequential progressive leukoplakia and oral cancer: drivers of oral tumorigenesis?
Source: Hum Mol Genet. 2014 Jan 8;23(10):2618–28. doi: 10.1093/hmg/ddt657 (PMC3990162; doi:10.1093/hmg/ddt657)
Supplement: Supplementary Data [file supp_ddt657_ddt657supp_table5.doc]

**Supplemental Table 5:** Non-progressive and progressive leukoplakia samples used for validation of aCGH data.

| **Patient ID** | **Sample Site** | **Histopathological Diagnosis** | **Age** | **Gender** | **Tobacco** |
| --- | --- | --- | --- | --- | --- |
| **NP 1** | Soft palate | Moderate dysplasia | 59 | M | No |
| **NP 2** | Tongue | Moderate dysplasia+hyperkeratosis | 33 | F | N/A |
| **NP 3** | Buccal mucosa | Moderate dysplasia | 44 | M | Yes |
| **NP 4** | Buccal mucosa | Mild dysplasia+hyperkeratosis | 50 | F | N/A |
| **NP 5** | Mandibular mucosa | Moderate dysplasia | 59 | F | N/A |
| **NP 6** | Lateral tongue | Moderate dysplasia+hyperkeratosis | 81 | F | N/A |
| **NP 7** | Lateral tongue | Mild dysplasia+hyperkeratosis | 62 | M | N/A |
| **NP 8** | Lateral pharyngeal wall | Severe dysplasia | 66 | M | Yes |
| **NP 9** | Buccal mucosa | Mild dysplasia | 61 | F | Yes |
| **NP 10** | Tongue | Severe dysplasia | 49 | M | No |
| **NP 11** | FOM | Mild dysplasia+hyperkeratosis | 53 | F | Yes |
| **NP 12** | Soft palate | Mild dysplasia+hyperkeratosis | 66 | M | N/A |
| **NP 13** | Tongue | Mild dysplasia | 51 | M | N/A |
| **NP 14** | FOM | Moderate dysplasia+hyperkeratosis | 50 | F | N/A |
| **NP 15** | Mand mucosa/FOM | Moderate dysplasia | 67 | M | N/A |
| **NP 16** | Mandibular mucosa | Moderate dysplasia+hyperkeratosis | 51 | M | N/A |
| **NP 17** | Buccal mucosa | Mild dysplasia | 39 | F | N/A |
| **NP 18** | Lateral tongue | Mild dysplasia+hyperkeratosis | 56 | M | N/A |
| **NP 19** | Buccal mucosa | Mild dysplasia+hyperkeratosis | 69 | M | Yes |
| **NP 20** | Tongue | Moderate dysplasia+hyperkeratosis | 37 | M | N/A |
| **NP 21** | Tongue | Moderate dysplasia+hyperkeratosis | 65 | F | No |
| **PG 1a** | Tongue | Dysplasia | 81 | M | No |
| **PG1b** | Tongue | OSCC | 81 | M | No |
| **PG2a** | Tongue | Dysplasia | 56 | M | Yes |
| **PG2b** | Tongue | OSCC | 56 | M | Yes |
| **PG3a** | Tongue | Dysplasia | 71 | F | Yes |
| **PG3b** | Tongue | OSCC | 71 | F | Yes |
| **PG4a** | Alveolar | Dysplasia | 74 | M | Yes |
| **PG4b** | Alveolar | OSCC | 74 | M | Yes |
| **PG5a** | FOM | Dysplasia | 56 | M | Yes |
| **PG5b** | FOM | OSCC | 56 | M | Yes |
| **PG6a** | Tongue | Dysplasia | 65 | F | Yes |
| **PG6b** | Tongue | OSCC | 65 | F | Yes |
| **PG7a** | Tongue | Dysplasia | 61 | M | Yes |
| **PG7b** | Tongue | OSCC | 61 | M | Yes |
| **PG8a** | Tongue | Dysplasia | 74 | F | No |
| **PG8b** | Tongue | OSCC | 74 | F | No |
| **PG9a** | Tongue | Dysplasia | 75 | M | Yes |
| **PG9b** | Tongue | OSCC | 75 | M | Yes |
| **PG10a** | FOM | Dysplasia | 71 | M | Yes |
| **PG10b** | FOM | OSCC | 71 | M | Yes |
| **PG11a** | Tongue | Dysplasia | 71 | M | Yes |
| **PG11b** | Tongue | OSCC | 71 | M | Yes |
| **PG12a** | Tongue | Dysplasia | 58 | M | Yes |
| **PG12b** | Tongue | OSCC | 58 | M | Yes |
| **PG13a** | FOM | Dysplasia | 64 | M | Yes |
| **PG13b** | FOM | OSCC | 64 | M | Yes |
| **PG14a** | Tongue | Dysplasia | 71 | M | Yes |
| **PG14b** | Tongue | OSCC | 71 | M | Yes |

NP: non-progressive leukoplakia; PG: progressive leukoplakia; FOM: floor of mouth

M: male; F: female; N/A: data not available.
