# Supplementary material for: Recurrent genomic alterations in sequential progressive leukoplakia and oral cancer: drivers of oral tumorigenesis?
Source: Hum Mol Genet. 2014 Jan 8;23(10):2618–28. doi: 10.1093/hmg/ddt657 (PMC3990162; doi:10.1093/hmg/ddt657)
Supplement: Supplementary Data [file supp_ddt657_ddt657supp_table6.doc]

**Supplemental Table 6.** List of 14 genes represented by several probes across the array, which were amplified or deleted in serial progressive leukoplakia lesions and paired OSCCs, but not in non-progressive samples. Data were normalized against Promega normal gDNA.

6 of these 14 genes (gene symbols in bold) were selected for validation by RQ-PCR.

| **Cytoband** | **Ref Seq_Genes**  **Gene symbol** | **Gene Name** | **Gene Function** | **Involvement in Cancer** |
| --- | --- | --- | --- | --- |
| **GAINS** | **AMPLIFIED** |  |  |  |
| **1p35.1** | **KHDRBS1** | KH domain containing, RNA binding, signal transduction associated 1(p62,Sam68) | DNA, RNA, and protein binding, SH3/SH2 adaptor activity, transcription repressor activity | Duran A et al 2008 Cancer Cell (The signaling adaptor p62 is an important NF-kappaB mediator in tumorigenesis). Lazer G et al 2007 Cell Signal. |
| **1q32.1** | CAMSAP1L1 | calmodulin regulated spectrin-associated protein (KIAA1078) | unknown | KIF14 (same region) is a candidate oncogene in the 1q minimal region of genomic gain in multiple cancers. Corson TW et al 2005 Oncogene. |
| **1q42.12** | **PARP1** | poly (ADP-ribose) polymerase family, member 1 | Involved in the base excision repair (BER) pathway, by catalyzing the poly(ADP-ribosyl)ation of a limited number of acceptor proteins involved in chromatin architecture and in DNA metabolism. This modification follows DNA damages and appears as an obligatory step in a detection/signaling pathway leading to the reparation of DNA strand breaks | Zaremba T et al 2009. |
| **2p14** | **RAB1A** | RAB1A, member RAS oncogene family isoform 1 | Probably required for transit of protein from the ER through Golgi compartment. Binds GTP and GDP and possesses intrinsic GTPase activity | Tongue; Shimada K et al 2005 BJ Cancer (found a high prevalence of Rab1A-overexpression not only in TSCCs (98%) but also in premalignant lesions (93%). |
| **5q31.3** | **HBEGF** | heparin-binding EGF-like growth factor | epidermal growth factor receptor binding.growth factor activity (EGFR ligand) | Chechlinska M et al 2009;Bos PD et al 2009 Nature. |
| **5q31.2** | **PAIP2** | poly(A) binding protein interacting protein 2 | translation repressor activity (interacts with VEGF mRNA - prognostic value in H&N SCC ) | Onesto C et al 2006 (H&N cancer; Vascular endothelial growth factor-A and Poly(A) binding protein-interacting protein 2 expression in human head and neck carcinomas: correlation and prognostic significance). Onesto C et al 2004. |
| **7p13** | TBRG4 | transforming growth factor beta regulator 4 | protein kinase activity | No reports |
| **10q24.32** | **NPM3** | nucleophosmin/nucleoplasmin 3 | nucleic acid binding, protein binding, rRNA processing and transcription | Two genetic pathways, t(1;10) and amplification of 3p11-12, in myxoinflammatory fibroblastic sarcoma, haemosiderotic fibrolipomatous tumour, and morphologically similar lesions - J. Pathol. 2009 Apr;217(5):716-27. |
| **11q13.4** | CHRDL2 | chordin-like 2, breast tumor novel factor 1 | cell differentiation, cartilage development, multicellular organismal development | BNF-1, a novel gene encoding a putative extracellular matrix protein, is overexpressed in tumor tissues. Gene 2003 Jun 5;311:105-10. |
| **14q12** | IRF9 | interferon regulatory factor 9 | protein binding, transcription factor activity | Expression of protein mediators of type I interferon signaling in human squamous cell carcinoma of the skin. Cancer Epidemiol. Biomarkers Prevention 2000 Sep;9(9):993-7. PTF1alpha/p48 transcription factor couples proliferation and differentiation in the exocrine pancreas [corrected]. Gastroenterology. 2004 Sep;127(3):937-49. |
| **14q12** | REC8 | REC8 homolog (yeast) | protein binding, male meiosis I, spermatogenesis | Upregulation of meiosis-specific genes in lymphoma cell lines following genotoxic insult and induction of mitotic catastrophe. BMC Cancer 2006 Jan 9;6:6. |
| **14q12** | GMPR2 | guanosine monophosphate reductase 2 | GMP reductase activity, metal ion binding, oxidoreductase activity | Cloning and functional characterization of GMPR2, a novel human guanosine monophosphate reductase, which promotes the monocytic differentiation of HL-60 leukemia cells. |
| **14q32.12** | BTBD7 | BTB (POZ) domain containing 7 | protein binding | FUP1, a gene associated with hepatocellular carcinoma, stimulates NIH3T3 cell proliferation and tumor formation in nude mice. Biochem Biophys Res Commun. 2001 Sep 7;286(5):1033-8 |
| **22q12.3** | FBXO7 | F-box protein 7 | Protein binding, ubiquitin-protein ligase activity. This gene encodes a member of the F-box protein family which is characterized by an approximately 40 amino acid motif, the F-box. The F-box proteins constitute one of the four subunits of the ubiquitin protein ligase complex called SCFs (SKP1-cullin-F-box), which function in phosphorylation-dependent ubiquitination. The F-box proteins are divided into 3 classes: Fbws containing WD-40 domains, Fbls containing leucine-rich repeats, and Fbxs containing either different protein-protein interaction modules or no recognizable motifs. The protein encoded by this gene belongs to the Fbxs class and it may play a role in regulation of hematopoiesis | Fbxo7 gets proactive with cyclin D/cdk6. Cell Cycle. 2006 Feb;5(3):279-82. Epub 2006 Feb 8 |
| **LOSSES** | **DELETED** |  |  |  |
| **8p23.2** | CSMD1 | CUB and Sushi multiple domains 1 | protein biding | Beuten J et al 2009; Sheffer M et al 2009; Kuo KT et al 2009. |
| **18q21.1** | MYO5B | MYO5B | ATP, actin, calmodulin, nucleotide biding | Kuang SQ et al 2008. |
