# Supplementary material for: Recurrent genomic alterations in sequential progressive leukoplakia and oral cancer: drivers of oral tumorigenesis?
Source: Hum Mol Genet. 2014 Jan 8;23(10):2618–28. doi: 10.1093/hmg/ddt657 (PMC3990162; doi:10.1093/hmg/ddt657)
Supplement: Supplementary Data [file supp_ddt657_ddt657supp_table7.doc]

**Supplemental Table 7**. Master Mix for Multiplex RQ-PCR using TaqMan Probes. RQ-PCR was performed using a standard program on the ABI 7900 (standard quantification method). The reference assay was labeled with 5’VIC fluorescent dye and TAMRA quencher.

| **Reaction mix component** | **Volume (μL)*** |
| --- | --- |
| 2X TaqMan Genotyping Master Mix | 5.0 |
| TaqMan Copy Number Assay, 20X (Gene)+ | 0.5 |
| TaqMan Copy Number Reference Assay | 0.5 |
| Nuclease-free water | 2.0 |
| **Total Volume** | **8.0** |

* 2 μL of genomic DNA (Diluted to 25 ng/μL) was added, thus 50ng of total DNA per each well, bringing the final volume to 10 μL. + Each primer for each gene was labeled with the 5’FAM fluorescent dye only (no quencher).
